# Maintenance of muscle mass, fiber size, and contractile function in mice lacking the Z-disc protein myotilin

**DOI:** 10.3109/03009730903276399

**Published:** 2009-12-08

**Authors:** Julien Ochala, Olli Carpén, Lars Larsson

**Affiliations:** ^1^Department of Clinical Neurophysiology, Uppsala University HospitalSweden; ^2^Department of Pathology, University of Turku and Turku University HospitalFinland; ^3^Center for Development and Health Genetics, The Pennsylvania State University, University ParkUSA

**Keywords:** Muscle, myotilin, single membrane permeabilized muscle fiber, telethonin

## Abstract

**Background:**

Myofibrillar myopathies constitute a rare group of congenital neuromuscular disorders, frequently associated with mutations in Z-disc proteins such as myotilin. Myotilin location and interactions with other Z-disc proteins are clearly defined, but its role in the regulation of muscle structure and function remains unknown. The present study aims at investigating this specific role of myotilin.

**Methods:**

Skeletal and cardiac muscles were collected from adult mice with a targeted deletion of myotilin (myo^-/-^) and wild-type animals (myo^+/+^).

**Results and conclusion:**

Similar skeletal and cardiac muscle weights were observed in myo^-/-^ and myo^+/+^ mice. At the muscle cell level, the size and force production of single membrane permeabilized fibers were identical between myo^-/-^ and myo^+/+^ rodents. Thus, myotilin does not have a significant influence on muscle mass, muscle fiber size, or regulation of muscle contraction. Alternatively, compensatory over-expressions of other elements including proteins from the same subfamily, or Z-disc proteins such as telethonin, or intermediate filaments may compensate for the lack of myotilin.

## Introduction

Myofibrillar myopathies are a group of rare congenital neuromuscular disorders (1). Clinical phenotypes of these particular diseases are remarkably homogeneous, consisting of slowly progressive proximal, distal, or both proximal and distal limb muscle weakness, occasionally accompanied by cardiac dysfunction. Histopathological characteristics include alterations of the myofibrillar architecture via Z-disc streaming and accumulation of Z-disc-related filamentous material (1,2). These myopathies are related to mutations in six different genes encoding sarcomeric Z-disc elements and related proteins, i.e. desmin (3), myotilin (4), αB-crystallin (5), Z-band alternatively spliced PDZ motif containing protein (ZASP) (6), filamin C (7) and four-and-a-half-LIM protein 1 (FHL1) (8), with myotilin defects being among the most common.

**Figure 1.**
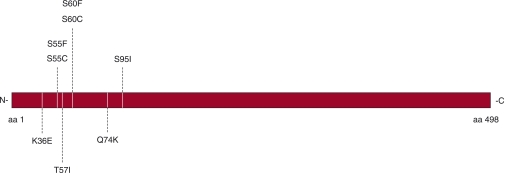
Schematic picture of the myotilin protein and the known mutations.

In 2000, the first myotilin mutation, a T57I missense defect, was reported in a large family with clinical symptoms consistent with autosomal dominant limb-girdle muscular dystrophy (LGMD) (9). At the age of 30 years, these patients suffered from proximal leg and arm muscle weakness, and a later progressive involvement of distal muscles occurred. Five additional myotilin mutations were subsequently identified. Histopathological analysis showed muscle fibers with disarrayed myofibrils (4). Normal striation pattern was occasionally replaced by widened and streaming Z-lines or rod-like structures that were not nemaline rods but rather streaks of dense material from Z-discs (4,9). Other cells exhibited large hyaline structures consisting of compacted fragmented filaments (4,9). All five myotilin defects had, therefore, Z-disc alterations, allowing an enhanced diagnostic precision, and this disorder now belongs to the myofibrillar myopathy group (4). To date, known myotilin mutations are heterozygous missense defects leading to point amino acid changes (Figure 1).

**Figure 2.**
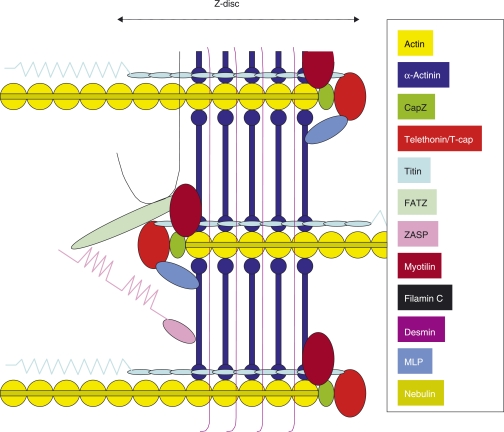
Schematic representation of the Z-disc in the myofibril. It should be noticed that myopalladin does not appear because its location is still in debate.

Myotilin is a 57-kDa protein composed of 498 amino acids, highly expressed in skeletal muscle and moderately in heart (10). This protein contains a serine-rich N-terminal region (amino acids 28 to 124) that also comprises a hydrophobic stretch (residues 57 to 79), two Ig-like domains (amino acids 252 to 341 and 351 to 441), and a C-terminal tail (10). As shown in Figure 2, myotilin is part of the Z-disc and interacts with several other proteins including α-actinin (11), actin (12), filamin C (13), FATZ-1, and FATZ-2 (14). In spite of all detailed information regarding myotilin structure and its localization in the Z-disc, there are no data on its physiological role in the sarcomere. The present study aimed at investigating this particular aspect by comparing mice having a targeted deletion of myotilin (myo^-/-^) with wild-type animals (myo^+/+^). Considering that the Z-disc proteins play pivotal roles in the assembly organization of the myofibrils and force generation (15), we hypothesized that the regulation of size and contractility are disrupted in myotilin-deficient muscle cells.

## Material and methods

### Animals

Four wild-type animals (myo^+/+^) and four myotilin knock-out mice (myo^-/-^) were included in the analyses (SV/129 strain, age 3 months). For a complete description of the *myo*^-/-^ mice, please see Moza et al. (16). Muscles (heart, right soleus, gastrocnemius, plantaris, tibialis anterior, and Extensor digitorum longus, EDL) of animals were dissected after cervical dislocation. All procedures involving animal care and handling were performed according to institutional guide-lines and were reviewed and approved by the local Ethical Committee on Animal Research.

### Muscle samples and permeabilization of fibers

Heart, gastrocnemius, plantaris, tibialis anterior, and EDL were frozen in liquid nitrogen-chilled propane and stored at −80°C. Soleus muscles were placed in relaxing solution at 4°C, and bundles of ∼50 fibers were dissected free and then tied with surgical silk to glass capillary tubes at slightly stretched lengths. The muscle bundles were then treated with skinning solution (relaxing solution containing glycerol; 50:50 v/v) for 24 hours at 4°C, after which they were transferred to −20°C. The muscle bundles were treated with sucrose, a cryo-protectant, within 1–2 weeks for long-term storage (17). After the sucrose treatment, muscle bundles were detached from the capillary tubes and snap-frozen in liquid nitrogen-chilled propane and stored at −160°C.

### Single muscle fiber experimental procedure

On the day of an experiment, a fiber segment 1–2 mm long was left exposed to the experimental solution between connectors leading to a force transducer (model 400A; Aurora Scientific) and a lever arm system (model 308B; Aurora Scientific) (18). The total compliance of the attachment system was carefully checked and remained similar for all the single muscle fibers tested (5% ± 0.5% of the fiber length). The apparatus was mounted on the stage of an inverted microscope (model IX70; Olympus). While the fiber segments were in relaxing solution, the sarcomere length was set to 2.65–2.75 µm by adjusting the overall segment length (19). The diameter of the fiber segment between the connectors was measured through the microscope at a magnification of ×320 with an image analysis system prior to the mechanical experiments. Fiber depth was measured by recording the vertical displacement of the microscope nosepiece while focusing on the top and bottom surfaces of the fiber. The focusing control of the microscope was used as a micrometer. Fiber cross-sectional area (CSA) was calculated from the diameter and depth, assuming an elliptical circumference, and was corrected for the 20% swelling that is known to occur during skinning (18). At 15°C, maximal force generation was calculated as the difference between the steady-state isometric force in activating solution and the resting force measured in the same segment while in the relaxing solution. Maximal force was adjusted for CSA and termed specific force. In addition, during steady-state isometric force production, a slack by 20% of the original fiber length was rapidly introduced (within 1–2 ms) at one end of the fiber, resulting in a rapid reduction of force to near zero. This was followed by a brief period of unloaded shortening (20 ms), after which the preparation was quickly restretched to its original length and the force was recovered to its original steady-state value. As described previously (20), the rate of force development (k_tr_) was estimated by linear transformation of the half-time of force redevelopment (t_1/2_) (21).

Relaxing and activating solutions contained (in mM) 4 Mg-ATP, 1 free Mg^2+^, 20 imidazole, 7 EGTA, 14.5 creatine phosphate, and KCl to adjust the ionic strength to 180 mM. The pH was adjusted to 7.0. The concentrations of free Ca^2+^ were 10^-9^ M (relaxing solution) and 10^-4.5^ M (activating solution), expressed as pCas (i.e. -log [Ca^2+^]). Apparent stability constants for Ca^2+^-EGTA were corrected for temperature (15°C) and ionic strength (180 mM). The computer program of Fabiato (22) was used to calculate the concentrations of each metal, ligand, and metal-ligand complex.

### Statistical analysis

Sigma Stat software (Jandel Scientific) was used to generate descriptive statistics. Data are presented as means ± standard error of the means (SEMs). Given the small number of fibers expressing the fast myosin heavy chain isoforms in the soleus muscles, comparisons were restricted to fibers expressing the slow myosin heavy chain isoform (type I). For all parameters, the unpaired *t* test was applied.

## Results and discussion

**Figure 3.**
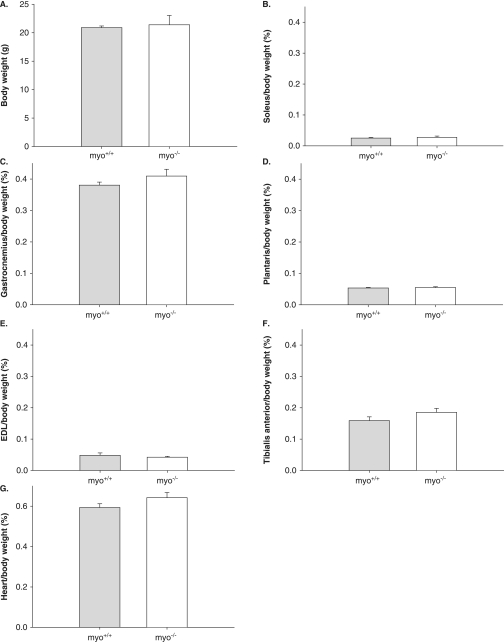
A: Body-weights, B: Soleus/body-weight, C: Gastrocnemius/body-weight, D: Plantaris/body-weight, E: EDL/body-weight, F: Tibialis anterior/body-weight, and G: Heart/body-weight of myo^-/-^ (white bars) and myo^+/+^ (gray bars) mice. All values are means ± SEMs.

**Figure 4.**
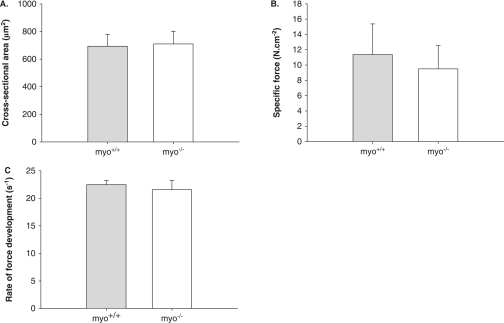
A: Cross-sectional area (CSA), B: Specific force, and C: Rate of force development (k_tr_) of membrane permeabilized fibers from myo^-/-^ (*n* = 28, white bars) and myo^+/+^ (*n* = 29, gray bars) mice. All values are means ± SEMs.

The weights of various fast- and slow-twitch distal hind limb muscles and the heart were unchanged (Figure 3). Such preservation is in accordance with data from a previous work in the same murine model (16). At the muscle cell level, the ‘skinned’ muscle fiber preparation was used, i.e. regulation of muscle contraction and muscle fiber size was measured at a fixed sarcomere length in single membrane permeabilized fiber segments. This technique allows detailed analyses in a muscle cell with an intact myofilament lattice, i.e. without the confounding effects of nerves, excitation-contraction coupling, fiber architecture, and intercellular connective tissue. Hence, after permeabilization, soleus muscle fibers were isolated from the bundles and mounted for analysis of cross-sectional area (CSA) at a fixed sarcomere length, maximal force production normalized to CSA (specific force), and rate of force development (k_tr_). A total of 57 fibers were included in the analyses. CSA, specific force, and k_tr_ were identical in fibers from myo^-/-^ and myo^+/+^ mice (Figure 4).

This was an unexpected observation that falsified our original hypothesis, indicating that myotilin does not play an important role in regulating or maintaining muscle structure and function. However, it cannot be ruled out that other proteins functionally compensate for the myotilin-deficient mice. Myotilin is part of a small subfamily of proteins, also including palladin and myopalladin (10). These functionally redundant proteins exist in all striated muscles, and their expression may be sufficient to overcome the lack of myotilin. Another obvious potential candidate for compensatory functions is the major component of the Z-disc, α-actinin (11). The 103-kDa protein α-actinin plays a key role in mediating, integrating, and preserving Z-disc interconnections as attested by the muscle disruption, dysfunction, and rapid death when it is lacking (23). However, the contents of α-actinin and other important Z-disc proteins including FATZ (34 kDa), ZASP (32/78 kDa), and myopalladin (145 kDa) are not affected in myo^-/-^ mice (16). ZASP (Z-band alternatively spliced protein) directly interacts with α-actinin, but it is neither required for muscle development nor Z-disc maturation, solely necessary for Z-disc integrity during contraction (24). Myopalladin interacts with nebulin and α-actinin and has a critical role in the assembly of the Z-disc by directly tethering nebulin and indirectly titin via α-actinin (25). Myopalladin over-expression leads to Z-disc disruption and myofibrillar disassembly (26).

The 19-kDa Z-disc protein, telethonin, also termed T-cap, is over-expressed in myotilin-deficient mice, both at the mRNA and protein levels (16). Telethonin controls myofibrillogenesis, and its over-expression in myo^-/-^ muscles may, consequently, reflect a compensatory mechanism, counteracting the weakened structure of the Z-disc (16). Furthermore, as shown in Figure 2, telethonin binds to the giant protein titin (3300 kDa) at the Z-disk periphery, leading to an increased Z-disk resistance to mechanical stress (27). Telethonin is also one of the mechano-sensing proteins in the sarcomere, and it responds to mechanical stretch stimuli by triggering subsequent downstream signaling for muscle cell growth and survival (28). Its up-regulation in muscles from myo^-/-^ mice has been suggested to represent an active response to modified mechano-sensing (16).

In addition to telethonin, the muscle LIM protein (MLP) may well be up-regulated in myotilin-deficient mice. MLP is a Z-disc protein with two LIM domains binding to α-actinin, telethonin, calcineurin, and β-spectrin (25). MLP maintains normal muscle characteristics and has both structural and gene-regulatory roles (29). MLP knock-out mice are characterized by muscle fiber atrophy and shorter resting sarcomere length (29). In addition, MLP mediates mechanical stress and is a critical mechano-sensor in the sarcomere (25). Apart from telethonin and MLP, the intermediate filament desmin may also be over-expressed. Desmin is a 52-kDa protein, linking the costameres to the Z-disc and interactions with e.g. nebulin, β-spectrin, ankyrins, calpain-3, synemin, and syncoilin (25). Desmin knock-out animals exhibit normal muscle development but have pronounced multisystem disorder involving cardiac, skeletal, and smooth muscle systems (30).

In conclusion, in the present study, results showed 1) unchanged skeletal and cardiac muscle masses in mice lacking myotilin when compared with wild-type animals; and 2) similar fiber cross-sectional area and force production between myo^-/-^ and myo^+/+^ mice. Consequently, one may suggest that the Z-disc protein myotilin does not play any important role in regulating or preserving muscle mass and contractility. Nevertheless, it should be noticed that in myo^-/-^ animals, up-regulation of telethonin and probably other proteins including those from the same subfamily may functionally compensate for the lack of myotilin.
